# Translating Metabolic Interventions into Breast Cancer Therapy: A Comprehensive Review

**DOI:** 10.3390/life15101634

**Published:** 2025-10-20

**Authors:** Luxi Chen, Stephen L. Shiao, Yuan Yuan

**Affiliations:** 1 Department of Medicine, Samuel Oschin Comprehensive Cancer Institute, Cedars-Sinai Medical Center, Los Angeles, CA 90048, USA; luxi.chen@cshs.org; 2 Department of Radiation Oncology, Cedars-Sinai Medical Center, Los Angeles, CA 90048, USA; stephen.shiao@cshs.org

**Keywords:** breast cancer, metabolic syndrome, signaling pathways, tumor microenvironment, cancer therapies

## Abstract

Breast cancer remains a leading cause of morbidity and mortality in women worldwide. Despite significant advances in targeted therapies, therapeutic resistance, metabolic toxicities, and disease recurrence continue to limit long-term efficacy. Metabolic syndrome is a major epidemiologic risk factor for the development of breast cancer, with metabolic dysregulation strongly linked to tumor progression, recurrence, and mortality. Crosstalk between insulin and insulin-like growth factor (IGF) signaling and oncogenic pathways such as PI3K/AKT/mTOR provides a mechanistic basis for these associations, highlighting the interplay between metabolism and tumor biology. Given this context, anti-diabetic and anti-obesity agents are being investigated as novel therapeutic strategies in breast cancer. Beyond their established metabolic benefits, these agents can directly modulate tumor cell growth, immune responses, and signaling pathways central to breast cancer pathogenesis. In this review, we summarize the current knowledge on the intersection of metabolic dysregulation and breast cancer as well as critically evaluate preclinical and clinical evidence supporting the use of metabolic therapies in this space.

## 1. Introduction

Breast cancer is the most frequently diagnosed malignancy and remains the leading cause of cancer-related mortality among women worldwide, with an estimated 2.3 million new cases and 665,684 deaths reported in 2022 [1]. Established breast cancer risk factors include age, genetic predisposition, hormonal and reproductive history, obesity, and lifestyle factors such as diet and physical inactivity [2]. Notably, a recent meta-analysis reported that higher dietary intake of essential fatty acids may be associated with an increased risk of breast cancer, highlighting the complex contribution of metabolic and nutritional factors to disease development [3]. While there has been remarkable progress in the treatment of breast cancer in the last decade, resistance to therapy and recurrence remain significant challenges.

The phosphatidylinositol 3-kinase (PI3K)/AKT/mammalian target of rapamycin (mTOR) pathway is a crucial signal transduction system that is aberrantly activated in breast cancer, mediating cell proliferation, survival, metabolism, and metastasis [4,5]. Targeting this signaling pathway has resulted in major breakthroughs in the treatment landscape of metastatic hormone receptor (HR)-positive breast cancer. As such, many Phase III clinical trials have led to the FDA approval of PI3K/AKT/mTOR inhibitors including alpelisib, capivasertib, inavolisib, and everolimus [6,7,8,9]. Utilization of these inhibitors has resulted in significantly prolonged progression-free survival and/or overall survival in breast cancer patients. However, given the important role of the PI3K/AKT/mTOR pathway in normal cell metabolism, major on-target, off-tumor side effects, particularly hyperglycemia and hyperlipidemia, have impaired wide adaptation of these inhibitors in clinical practice [10].

Obesity and type 2 diabetes mellitus (T2DM) are independent risk factors for the development of various cancers, including breast, colorectal, pancreatic, esophageal, gastric, and endometrial cancers [11,12]. Increased body weight and obesity augment the risk of triple negative breast cancer (TNBC) in premenopausal women by 67% and premenopausal luminal B breast cancer by 73%, in contrast to women of normal weight [13,14]. In addition, obese women with breast cancer have an approximately 30% increased risk of cancer recurrence or death compared to those with normal weight [15]. Both obesity and T2DM are associated with a higher risk of postmenopausal breast cancer [16]. Several studies have shown an increased breast cancer specific mortality in patients with metabolic syndrome, likely due to crosstalk between the insulin-like growth factor (IGF) family and signaling pathways that drive tumorigenesis, including the PI3K/AKT/mTOR pathway [17,18].

Given the above, anti-diabetic and anti-obesity medications such as metformin and glucagon-like peptide-1 receptor agonists (GLP-1RAs) have recently emerged as potential therapeutic candidates in the treatment of breast cancer [19]. By simultaneously modulating metabolic pathways and directly influencing tumor and immune cell signaling, these agents hold the potential to complement existing targeted therapies, reduce toxicities, and improve long-term outcomes in breast cancer patients. This review will explore the mechanistic underpinnings, preclinical evidence, and emerging clinical data supporting the role of metabolic interventions in redefining the landscape of breast cancer treatment.

## 2. Methodology

We conducted a targeted search of PubMed, ClinicalTrials.gov, and Google Scholar using combinations of relevant keywords (“metabolic syndrome,” “breast cancer,” “insulin signaling,” “tumor signaling pathways,” “metformin,” “GLP-1 receptor agonists,” “SGLT2 inhibitors”). Both preclinical and clinical studies were considered, with emphasis on high-impact epidemiological analyses, seminal mechanistic investigations, and Phase II–III clinical trials with defined endpoints. ClinicalTrials.gov was systematically reviewed to capture ongoing and completed studies of metabolic interventions in breast cancer, and reference lists of key publications were screened to identify additional relevant reports. This strategy enabled a rigorous synthesis of current evidence across epidemiologic, mechanistic, and translational domains.

## 3. Metabolic Syndrome and Breast Cancer

### 3.1. Epidemiological Evidence

Multiple studies have demonstrated a significant association between the development of breast cancer and metabolic syndrome, a condition characterized by central obesity, dyslipidemia, hypertension, and impaired glucose tolerance [20,21]. In postmenopausal women, obesity has consistently been shown to increase the rate of breast cancer by 30–50% [22]. Further investigation on breast cancer survival and mortality indicate that adiposity is associated with lower survival rates and higher likelihood of recurrence, irrespective of menopausal status, tumor stage, and treatment [23]. In fact, breast cancer patients with a body mass index (BMI) ≥ 40.0 kg/m^2^ not only have a mortality rate three times higher compared to those with BMI < 20.5 kg/m^2^ but is also associated with poorer differentiation, larger tumor size, and increased lymph node invasion frequency [24].

Recently, the Women’s Health Initiative (WHI) clinical trials showed that a higher metabolic syndrome score, which included factors such as high waist circumference and history of T2DM, hypertension, and/or high cholesterol, in postmenopausal breast cancer patients was significantly associated with poorer prognosis and higher mortality rates [20]. In the WHI cohort of 544 women with TNBC, after a median follow-up of 8.3 years, those with three or four risk factors had more than double the risk of breast cancer–specific mortality (HR 2.05; 95% CI 0.94–4.47; trend *p* = 0.114, not statistically significant) and a significantly higher risk of overall mortality (HR 2.13; 95% CI 1.22–3.71; trend *p* = 0.006) [25]. Additionally, a meta-analysis demonstrated that T2DM was associated with an increased risk of both estrogen receptor-positive (ER+) (RR = 1.09, 95% CI: 1.00–1.20) and triple-negative breast cancer (RR = 1.41, 95% CI: 1.01–1.96) [26].

Racial and ethnic disparities further shape the epidemiological landscape of obesity and breast cancer risk, particularly in the United States. Obesity prevalence is disproportionately higher among Black women compared to other racial and ethnic groups, a difference attributed to a complex interplay of genetic, biochemical, behavioral, and socioeconomic factors. Recent analyses demonstrate that these disparities are not only widespread but also persist across age groups and geographic regions. For example, a national cohort study reported a significantly higher prevalence of both general and abdominal obesity among non-Hispanic Black women compared with non-Hispanic White women, findings that have important implications for breast cancer risk and outcomes in this population [27]. Moreover, epidemiologic data indicate that Black women experience higher rates of metabolic syndrome, which may contribute to the observed disparities in breast cancer incidence and prognosis [28]. These findings highlight the need to integrate racial and ethnic considerations into studies examining the metabolism–cancer interface, particularly when evaluating prevention and intervention strategies.

### 3.2. Obesity-Induced Estrogen Production

One of the principal mechanisms underlying the association between metabolic syndrome and the development of breast cancer is the obesity-driven increase in systemic estrogen levels. In postmenopausal women, adipose tissue becomes the predominant site of estrogen biosynthesis through the activity of the cytochrome P450 enzyme aromatase, which catalyzes the conversion of adrenal androgens, such as androstenedione and testosterone, into estrone and estradiol [29,30]. Elevated estrogen availability enhances estrogen receptor signaling, thereby stimulating breast epithelial proliferation and promoting tumorigenesis [31]. Notably, aromatase is expressed not only in peripheral adipose depots, such as breast, abdominal, thigh, and gluteal fat, but can also be upregulated within the tumor tissue itself, creating a localized estrogen-rich microenvironment that supports malignant cell growth [32].

### 3.3. Chronic Inflammation in Adipocytes

Beyond estrogen production, obesity induces chronic low-grade inflammation within adipose tissue, which contributes to tumorigenesis through multiple signaling pathways. Obese adipocytes secrete pro-inflammatory cytokines such as tumor necrosis factor-α (TNF-α) and interleukin-6 (IL-6), which activate the “obesity–inflammation–aromatase axis” [33,34]. These cytokines enhance transcription of the CYP19A1 gene, further increasing aromatase expression and thereby amplifying intratumoral estrogen biosynthesis [35]. This inflammatory milieu is accompanied by alterations in adipose-derived endocrine function.

Adipose tissue is now recognized as a metabolically active endocrine organ that secretes a wide range of bioactive molecules, collectively termed adipokines, which exert systemic effects on metabolism, immune function, and tumor biology [36]. Among these adipokines, leptin has been consistently implicated in breast cancer progression. Hyperleptinemia, commonly observed in obesity, promotes cell proliferation, angiogenesis, and resistance to apoptosis, largely through activation of the Janus kinase/signal transducers and activators of transcription (JAK/STAT), phosphatidyl inositol 3 kinase (PI3K)/AKT, and mitogen-activated protein kinase (MAPK) signaling pathways [37,38]. In contrast, the adipokine adiponectin appears to exert protective effects, with anti-proliferative, insulin-sensitizing, and anti-inflammatory properties. Low circulating adiponectin levels have been associated with increased breast cancer risk, more aggressive tumor phenotypes, and poorer clinical outcomes in studies [39,40]. Collectively, these mechanisms highlight the multifaceted contribution of obesity to breast cancer pathogenesis, through both endocrine and paracrine signaling networks that converge on estrogen metabolism, chronic inflammation, and adipokine imbalance.

### 3.4. Insulin Resistance and Breast Cancer Risk

Another hallmark of metabolic syndrome, insulin resistance, has been independently associated with increased breast cancer risk. The mechanistic basis for this relationship is thought to involve bidirectional crosstalk between estrogen signaling and the insulin/insulin-like growth factor-1 (IGF-1) axis [41]. Estrogen receptor α (ERα) activity can be modulated by insulin and IGF-1 through both genomic and non-genomic mechanisms, including phosphorylation of ERα and its coregulators via downstream PI3K/AKT and MAPK signaling cascades [42,43]. Estrogen enhances the expression and activity of key mediators of the insulin/IGF-1 pathway, thereby amplifying proliferative and anti-apoptotic signaling in breast epithelial cells [44]. This synergistic interplay promotes mitogenic signaling, metabolic reprogramming, and resistance to cell death, features that may contribute to the preferential association of T2DM with ER+ tumors. Moreover, hyperinsulinemia and elevated IGF-1 bioavailability in T2DM create a systemic environment that favors tumor initiation and progression via enhanced activation of mitogenic pathways [45]. Altogether, these findings support the hypothesis that metabolic- and hormone-driven signaling converge to increase susceptibility to ER+ breast cancer in women with T2DM.

### 3.5. Current Models to Study Metabolic Dysregulation in Breast Cancer

Complementary in vitro and in vivo systems are often employed to study metabolic dysregulation in breast cancer. Extensively characterized human breast cancer cell lines (i.e., ER+ MCF-7/T-47D; HER2+ SK-BR-3; TNBC MDA-MB-231/MDA-MB-436) enable controlled interrogation of glycolysis, oxidative metabolism, and lipid pathways [46]. To better replicate tumor architecture and nutrient–oxygen gradients, three-dimensional cultures and patient-derived organoid co-culture systems are increasingly employed to model cell–cell and cell–matrix interactions that influence metabolic flux, although variability in derivation, passaging, and microenvironmental fidelity remains a challenge [47]. For translational applications, animal models are commonly utilized. These include syngeneic murine tumors (e.g., 4T1, E0771) in immunocompetent hosts to study immune-metabolic crosstalk, genetically engineered mouse models and patient-derived xenografts to preserve oncogenic drivers and intratumoral heterogeneity, and obesity/insulin-resistance models to examine how adiposity, hyperinsulinemia, and dyslipidemia reprogram tumor and immune compartments [48].

## 4. IGF-1 Signaling in Breast Cancer

### 4.1. The IGF-1 Signaling Pathway

Insulin-like growth factor-1 (IGF-1) is a peptide hormone structurally related to insulin that exerts potent anabolic and mitogenic effects [49]. Its production is primarily regulated by growth hormone (GH) and represents a critical mediator of GH’s systemic and tissue-specific biological actions [50]. IGF-1 signaling is initiated through binding to the IGF-1 receptor (IGF-1R), a transmembrane tyrosine kinase receptor belonging to the same family as the insulin receptor. Upon ligand binding, IGF-1R undergoes autophosphorylation and recruits adaptor proteins such as insulin receptor substrates and SH2-domain containing proteins, which function as docking molecules to propagate downstream signaling [51]. Activation of IGF-1R triggers multiple intracellular cascades that regulate cell growth, survival, and metabolism.

Two major oncogenic pathways are preferentially activated: the PI3K/AKT/mTOR pathway and the Ras/Raf/MEK/extracellular signal-regulated kinase (ERK) cascade [52]. The PI3K/AKT/mTOR axis promotes protein synthesis, cell proliferation, and metabolic reprogramming, while simultaneously inhibiting apoptosis through phosphorylation of pro-apoptotic proteins and activation of anti-apoptotic members of the Bcl-2 family [49]. In parallel, Ras/Raf/MEK/ERK signaling drives cell cycle progression and enhances mitogenic activity, further contributing to tumor initiation and progression.

### 4.2. Effect of IGF-1 Signaling on Breast Cancer Biology

IGF-1 has long been implicated in mammary gland biology, where it plays a critical role in ductal morphogenesis, mammary gland development, and maintenance of epithelial cell homeostasis [53]. In breast cancer, aberrant activation of the IGF axis is frequently observed. Both preclinical and clinical studies demonstrate that the IGF-1R is overexpressed and hyperphosphorylated across multiple breast cancer subtypes, thereby providing a mechanistic basis for its oncogenic contribution [54]. Elevated circulating levels of IGF-1 and its primary binding protein, IGFBP-3, have been identified as independent risk factors for both breast cancer development and recurrence in the general population [55]. This association is particularly strong for ER+ tumors, irrespective of menopausal status, suggesting that IGF-1 synergizes with estrogen receptor signaling to drive tumor initiation and progression [55].

In addition, metabolic dysregulation and obesity appear to amplify IGF-1-mediated oncogenic effects. Increased BMI and associated dysmetabolic states have been shown to influence IGF-1 signaling, particularly in human epidermal growth factor receptor 2 (HER2)-positive patients, in which overweight status is correlated with enhanced IGF pathway activity and worse clinical outcomes [56]. IGF-1 may also function as a prognostic biomarker. Several studies have suggested that elevated circulating IGF-1 confers negative prognostic significance, both in unselected breast cancer populations [57] and in women undergoing endocrine therapy, where IGF-1 activation has been linked to therapeutic resistance and disease progression [57,58].

### 4.3. IGF-1 Dysregulation in Breast Cancer Treatment Resistance

Importantly, dysregulation of the IGF-1/IGF-1R axis has been increasingly implicated in therapy resistance in breast cancer. In ER+ disease, IGF-1R signaling exhibits extensive crosstalk with the ERα pathway, enhancing ligand-independent ER activation and promoting resistance to endocrine therapies such as tamoxifen and aromatase inhibitors [59]. Similarly, in HER2+ breast cancer, IGF-1R cooperates with HER2/ERBB2 signaling to sustain downstream PI3K/AKT and MAPK activation, thereby undermining the efficacy of HER2-targeted agents such as trastuzumab [60]. Beyond receptor crosstalk, IGF-1-mediated activation of survival pathways also contributes to resistance against chemotherapy and radiotherapy by promoting DNA damage repair, inhibiting apoptosis, and enhancing epithelial-to-mesenchymal transition, which fosters metastatic progression [61]. In summary, these findings demonstrate that IGF-1R signaling not only drives tumorigenesis but also serves as a critical mediator of therapeutic resistance across multiple breast cancer subtypes, highlighting the potential of targeting IGF-1R or its downstream pathways as a strategy to overcome resistance and improve patient outcomes.

## 5. Glucose Metabolism Reprograming in the Tumor Microenvironment

Glucose metabolism reprogramming exerts profound effects not only within breast cancer cells but also across the broader tumor microenvironment, where it influences the function of immune and stromal compartments, including T cells, macrophages, and cancer-associated fibroblasts (CAFs) [62]. Crosstalk among these cell types is largely mediated by key glucose metabolites such as lactate and pyruvate. Elevated intratumoral lactate accumulation and the resulting acidification suppress antitumor immunity, whereas reducing lactate levels has been shown to enhance immunosurveillance and improve responses to immunotherapies [63].

Lactate also functions as a critical immunomodulator, driving the polarization of macrophages from pro-inflammatory M1-like states to immunosuppressive M2-like phenotypes, while exerting context-dependent effects on T cell function [64]. In parallel, CAFs undergo a “reverse Warburg effect,” wherein they secrete lactate and pyruvate that are subsequently imported by adjacent cancer cells, fueling tumor proliferation and contributing to multidrug resistance [65,66]. Distinct metabolic programs further delineate T cell subsets: effector T cells rely on glycolysis and glutamine metabolism to support proliferation and differentiation, while regulatory T cells (Tregs) favor oxidative metabolism and display reduced glycolytic activity. This divergence may partly explain why TNBC tumors characterized by heightened glycolytic activity often demonstrate poor responsiveness to immune checkpoint blockade [63]. Together, these findings emphasize the central role of glucose metabolism in shaping tumor–immune–stromal interactions and suggest that targeting metabolic reprogramming may enhance antitumor immunity and improve responsiveness to existing therapies in breast cancer.

## 6. Targeting Glucose Metabolism in Breast Cancer

### 6.1. Metformin

Metformin, a first-line oral antidiabetic agent belonging to the biguanide class, is widely used for the management of T2DM due to its ability to reduce circulating glucose levels and enhance insulin sensitivity in peripheral tissues [67]. Over the past twenty years, a growing body of research has linked metformin use to lower rates of cancer incidence and mortality, sparking considerable interest in exploring its potential as an anticancer therapy [68,69]. At the systemic level, metformin reduces hepatic gluconeogenesis and consequently lowers circulating insulin concentrations, thereby attenuating activation of insulin- and IGF-1-dependent signaling cascades that are frequently co-opted in tumor growth [70]. At the cellular level, metformin has been shown to activate AMP-activated protein kinase (AMPK), a central metabolic sensor and tumor suppressor [71]. Elevated AMPK activity exerts downstream inhibitory effects on two critical oncogenic signaling axes, the PI3K/AKt/mTOR and Ras/Raf/MEK/ERK pathways, both of which regulate protein synthesis, cell cycle progression, and proliferation [72]. By dampening these anabolic and mitogenic signals, metformin can inhibit tumor cell growth and survival.

### 6.2. Metformin Preclinical Evidence

In preclinical models of breast cancer, metformin treatment has produced positive effects on dampening tumor progression. In a 2005 study by Anisimov et al., lifelong metformin administration (240 mg/kg body weight) starting at two months of age in transgenic FVB/N female mice with the HER-2/neu mammary cancer gene led to an approximate 8% increase in lifespan [73]. Although metformin did not reduce tumor incidence or the number of tumors (as all mice developed tumors), it significantly delayed tumor onset and reduced tumor size. Similarly, the Schedin laboratory demonstrated that in female Wistar rats (both with intact ovaries and those ovariectomized) fed a high-fat diet and treated with MNU to induce mammary tumors, an 8-week course of metformin (2 mg/mL) significantly lowered tumor volume [74,75]. In the ovariectomized group, metformin also reduced the presence of aromatase-positive macrophages at the tumor margins. Since aromatase converts androgens into estrogens, this finding suggests that metformin may suppress local estrogen production, potentially limiting the growth of ER-positive tumor cells [76]. Notably, metformin’s anticancer activity may extend beyond hormone-responsive cancers, as it also inhibited the growth of triple-negative breast tumors derived from MDA-MB-231 cells in BALB/c-nu nude mice [77].

### 6.3. Metformin Clinical Studies

Several clinical trials have investigated the use of metformin in combination with standard-of-care treatments in breast cancer. The Phase II NCT02028221 trial demonstrated that metformin intake in premenopausal women with a BMI ≥ 25 who were at risk for breast cancer resulted in favorable changes in measures of adiposity and borderline breast density reduction compared to placebo [78]. The meaningful reductions in central adiposity, as measured by waist circumference and waist-to-hip ratio, exemplify metformin’s potential as a metabolic intervention to modulate obesity-related breast cancer risk. Another study, the Phase II NCT01340300 clinical trial, investigated the effect of exercise ± metformin in patients with stage I-III breast cancer post adjuvant treatment. The trial reported that both exercise and metformin led to significantly reduced levels of fasting insulin and improvements in BMI compared to the control group (NCT01340300).

MA.32 is a Phase III randomized, double-blind, placebo-controlled international study designed to evaluate whether the addition of metformin to standard adjuvant therapy improved outcomes in patients with high-risk operable breast cancer (NCT01101438). The results demonstrated that addition of metformin to standard adjuvant therapy did not confer a significant improvement in invasive disease–free survival (IDFS) or other key breast cancer outcomes when compared to placebo [79]. Exploratory analyses from the trial raised the possibility of a clinically relevant effect of metformin in specific molecular subgroups. For instance, in HER2+ (ERBB2+) breast cancer, metformin use was associated with longer IDFS and overall survival, particularly among patients harboring at least one C allele of the rs11212617 single nucleotide variant (SNV). This observation is consistent with findings from the METTEN study, which reported a markedly higher pCR rate when metformin was added to neoadjuvant chemotherapy and HER2-targeted therapy in patients carrying the C allele (81.2% vs. 35.3% in non-carriers) [80].

The Phase II METEOR trial evaluated the combination of neoadjuvant metformin with aromatase inhibitor letrozole in postmenopausal women with ER+ breast cancer (NCT01589367) [81]. The trial reported an overall clinical response rate of 61.4% but did not reach statistical significance between the metformin + letrozole vs. placebo + letrozole cohort (66.7% versus 56.4%, *p* = 0.193). Though this difference lacked statistical significance, early reductions in Ki-67 at 4 weeks, particularly in the metformin group, was predictive of clinical response. Recently, the BREAKFAST Phase II trial investigated whether cyclic fasting-mimicking diets (FMD) with or without metformin can enhance the efficiency of neoadjuvant chemotherapy in patients with localized (Stages I–III, cT > 1 cm) TNBC (NCT04248998). Despite early termination due to adoption of chemoimmunotherapy as the new standard-of-care treatment, the trial reported an overall pCR rate of 56.6%, markedly higher than the historical range of 26–39% reported in prior Phase II/III neoadjuvant chemotherapy trials [82]. RNA sequencing of tumor samples uncovered that the patients who achieved pCR exhibited robust early infiltration of activated T cells and natural killer (NK) cells [83]. Concurrently, tumors demonstrated early downregulation of glycolysis and mitochondrial oxidative phosphorylation pathways. These findings suggest that metabolic reprogramming and immune activation underlie the enhanced response observed with FMD ± metformin.

### 6.4. SGLT2 Inhibitors

Sodium–glucose co-transporter 2 inhibitors (SGLT2i), also known as gliflozins, represent a relatively novel class of orally administered glucose-lowering agents that have demonstrated robust efficacy in the management of glycemic control. Mechanistically, SGLT2 blockade reduces renal glucose reuptake, induces glycosuria, and lowers plasma glucose levels through an insulin-independent mechanism, an important advantage in patients with impaired β-cell function or insulin resistance [84].

### 6.5. SGLT2i Preclinical Evidence

In breast cancer, SGLT2 expression has been detected in both primary human breast cancer tissue as well as cell lines via immunohistochemistry and RT-PCR [85,86]. Preclinical studies provide compelling evidence that SGLT2i can exert direct anti-tumor effects. Zhou and colleagues demonstrated that treatment with SGLT2i dapagliflozin and canagliflozin suppressed breast cancer cell proliferation both in vitro and in vivo using nude mouse xenograft models [87]. The inhibitory effects were linked to cell cycle arrest and induction of apoptosis, indicating that SGLT2 blockade disrupts essential tumor cell survival pathways. Furthermore, SGLT2i exposure modulated key signaling cascades, enhancing phosphorylation of AMP-activated protein kinase (AMPK), a central energy-sensing and tumor-suppressive kinase, while simultaneously reducing phosphorylation of 70 kDa ribosomal protein S6 kinase 1 (p70S6K1), a critical downstream effector of the mTOR pathway involved in protein synthesis and cell growth [87].

In another investigation, ipragliflozin was shown to exert a potent inhibitory effect on breast cancer cell proliferation. Specifically, treatment with ipragliflozin at concentrations ranging from 1 to 50 μM significantly and dose-dependently suppressed growth of the human ER+ breast cancer cell line, MCF-7 [88]. Importantly, this anti-proliferative effect was abrogated when SGLT2 expression was silenced via targeted knockdown, providing strong evidence that the observed growth inhibition is mediated through blockade of the SGLT2 transporter. Furthermore, SGLT2i can augment the therapeutic efficiency of cytotoxic chemotherapy. In a study by Akingbesote et al., dapagliflozin reduced tumor glucose uptake and enhanced the cytotoxic effects of paclitaxel by lowering circulating insulin, ultimately prolonging overall survival in the MMTV-PyMT murine model of breast cancer [89]. Interestingly, this benefit was not universally observed across all breast tumor subtypes. The enhanced response was restricted to tumors characterized by mutations occurring upstream of canonical insulin signaling pathways, such as those affecting receptor or proximal signaling components. In contrast, tumors harboring mutations downstream of the insulin signaling cascade, where signaling remains constitutively active irrespective of insulin levels, did not exhibit the same degree of chemosensitization.

### 6.6. SGLT2i Clinical Studies

Many ongoing clinical trials are investigating the combination of SGLT2i with standard-of-care anticancer therapies, including PI3K inhibitors (i.e., alpelisib), endocrine therapies (i.e., fulvestrant), and conventional chemotherapeutic regimens. The TIFA study is an ongoing multicenter, randomized Phase II clinical trial designed to assess the efficacy of SGLT2i canagliflozin in combination with standard-of-care treatment alpelisib plus fulvestrant in preventing PI3K inhibitor-associated hyperglycemia in patients with metastatic, PIK3CA-mutant, HR+HER2- breast cancer (NCT05090358). The primary endpoint is the proportion of patients who remain free from grade 3–4 hyperglycemia at 12 weeks. Key secondary endpoints extend beyond glycemic safety and include the overall response rate (ORR) and progression-free survival (PFS) at 6 and 12 months, providing insight into whether improved glycemic control translates into enhanced cancer-related efficacy. Furthermore, the study will evaluate adherence to alpelisib, indicating that effective mitigation of hyperglycemia likely leads to sustained drug exposure and optimization of therapeutic benefit.

Another clinical trial, NCT05989347, is a single-arm Phase I study that examines whether dapagliflozin, administered in conjunction with standard neoadjuvant therapies, can favorably alter metabolic markers of insulin resistance in women with early-stage HER2- breast cancer. Specifically, the study will assess changes in fasting plasma glucose and insulin levels as surrogate markers for insulin resistance. Additionally, it explores whether dapagliflozin can be safely integrated into multi-agent chemotherapy regimens without compromising tolerability. SGLT2i are also under investigation for their potential as cardioprotective agents. The multicenter, Phase III PROTECTAA trial aims to evaluate whether dapagliflozin can prevent anthracycline-related cardiotoxicity in non-diabetic patients with stage I–III invasive breast cancer (NCT06304857). The trial not only explores a novel strategy for mitigating a major treatment-limiting toxicity but also broadens the potential application of SGLT2i beyond their established roles in diabetes, heart failure, and chronic kidney disease.

### 6.7. Glucagon-like Peptide-1 Receptor Agonists

Glucagon-like peptide-1 (GLP-1) is an incretin hormone produced by intestinal L-cells in response to food intake, increasing insulin secretion and inhibiting glucagon release from the pancreas. However, therapeutic use of GLP-1 is limited by its short half-life due to rapid degradation by dipeptidyl peptidase-4 (DPP-4) [90]. GLP-1 receptor agonists (GLP-1RAs) were developed to mimic the action of native GLP-1 and engineered to resist DPP-4 degradation. As a result, they are highly efficacious and widely utilized for the management of T2DM [91]. In addition, GLP-1RAs have pleotropic effects on reducing plasma glucose, inducing weight loss, and immune modulation [92], resulting in their recent FDA approval for the treatment of obesity [93]. Recently, GLP-1RAs have garnered attention for their multifaceted roles beyond glycemic control, including in malignancy through modulating anti-tumor immunity and regulating signaling pathways involved in cancer cell growth and survival. In breast cancer, various GLP-1RAs have been shown to result in a decrease in tumor size, weight, and proliferation index [94].

### 6.8. GLP-1R Expression in Breast Cancer

Studies have demonstrated that GLP-1R is expressed on human breast cancer cell lines as well as primary breast cancer tissue. Iwaya et al. reported the expression of membrane-localized GLP-1R in human breast cancer tissue via immunohistochemical staining for GLP-1R and three human breast cancer cells lines (MCF-7, MDA-MB-231, and KPL-1) [95]. Among the breast cancer cell lines, MCF-7 cells, which display high estrogen-sensitive proliferation, had the highest *GLP-1R* gene expression as measured by quantitative real-time PCR (RT-PCR). In addition, transfection of MCF-7 cells using a GLP-1R-expressing lentiviral vector containing a Flag epitope tag showed exclusive GLP-1R expression to the breast cancer cell membrane rather than the cytosol or nucleus. Similarly, Tanaka et al. also confirmed that GLP-1R is expressed on MCF-7 cells via immunofluorescence analysis [96]. Another study showed that GLP-1R was widely expressed in many human breast cancer cell lines, including MCF-7, MDA-MB-231, BT483, MDA-MB-468, and ZR751, via RT-PCR and Western blot analyses [97].

### 6.9. Exendin-4

Exendin-4 (Ex-4) is a GLP-1RA that mimics the incretin hormone GLP-1, enhancing insulin secretion and exhibiting glucose-lowering effects [98]. Beyond its role in diabetes treatment, emerging evidence suggests that Ex-4 may influence tumor cell dynamics, including those of breast cancer. Fidan-Yaylali et al. demonstrated that Ex-4 significantly suppressed the proliferation of MCF-7 breast cancer cells by inducing apoptosis and inhibiting MCF-7 cell migration, invasion, and colony formation [99].

In a similar vein, Iwaya et al. showed that Ex-4 attenuated breast cancer cell proliferation in vitro in MCF-7 cells via inhibition of NF-*κ*B activation and subsequent target gene expression, including VEGF, IL-8, and COX-2 [95]. Treatment with Ex-4 dramatically reduced Ki-67 expression, a marker of cancer cell proliferation and cell cycle progression. Furthermore, Ex-4 treatment inactivated Akt and I*κ*B*α* phosphorylation in MCF-7 cells. Anti-carcinogenesis activity was also present in vivo, with significantly decreased tumor size observed in Ex-4-treated athymic nude mice transplanted with MCF-7 cells. Nine weeks after subcutaneous transplantation of MCF-7 cells, tumor size was nearly halved in Ex-4-treated mice compared to controls, while body weight and blood glucose levels remained unchanged. This suggests that Ex-4 suppresses tumor growth independently of its systemic metabolic effects.

### 6.10. Liraglutide

Preclinical studies evaluating the effects of liraglutide on breast cancer cells demonstrate that the second generation GLP-1RA exhibits both anti-proliferative and pro-proliferative properties. In one report, liraglutide demonstrated the ability to suppress proliferation and induce apoptosis in MCF-7 breast cancer cells [100]. Treatment with liraglutide resulted in decreased cell viability and increased apoptotic factors, such as cleaved-caspase-3. These effects were dose- and time-dependent, with significant changes observed at higher concentrations and prolonged exposure times. Mechanistically, liraglutide downregulates microRNA-27a (miR-27a), leading to the upregulation of AMP-activated protein kinase catalytic subunit α2 (AMPKα2), a tumor suppressor gene involved in cell cycle regulation and apoptosis. The modulation of miR-27a and AMPKα2 suggests a pathway through which liraglutide exerts its antiproliferative effects on estrogen receptor-positive breast cancer cells.

Similarly, Alanteet et al. showed that in models simulating obesity-associated breast cancer, liraglutide attenuated tumor cell proliferation by inhibiting the PI3K/Akt/mTOR signaling pathway [101]. Growth of liraglutide-treated MCF-7 cells incubated in conditioned media generated from human adipose-derived stem cells (ADSCs) significantly decreased by approximately 48% when compared to no treatment. When treated with liraglutide, MCF-7 cells from either lean ADSCs or obese ADSCs displayed significantly reduced protein levels of phosphorylated (p)-PI3K, p-Akt, and p-mTOR. Furthermore, the anti-proliferative effects exhibited by liraglutide were similar to those mediated by PI3K and mTOR inhibitors, LY294002 and rapamycin, respectively. Contrasting findings have emerged in studies involving human triple-negative breast cancer (TNBC) cell lines. In one report, a slightly higher concentration of liraglutide accelerated breast cancer progression in vitro and in vivo through the NOX4/ROS/VEGF signaling pathway after activation the GLP-1 receptor [97]. In vitro, liraglutide promoted the proliferation and migration ability of two TNBC cell lines, MDA-MB-231 and MDA-MB-468, by significantly increasing intracellular ATP. This effect was able to be reversed by GLP-1R inhibitor, Exendin.

### 6.11. Semaglutide

Semaglutide, a second-generation incretin analog, has recently been shown to exhibit anti-tumor effects in a murine model of TNBC. Intraperitoneal administration of semaglutide in BALB/c mice implanted with syngeneic 4T1 TNBC cells resulted in decelerated tumor growth, appearance, and progression [102]. Notably, semaglutide-treated mice had significantly smaller tumor diameters compared to the control group on day 14 after tumor induction (*p* = 0.011). The authors further explored its effects on anti-tumor immunity, demonstrating that semaglutide enhanced the activation and cytotoxicity of CD8+ T cells within the tumor microenvironment, modulated macrophage polarization towards a pro-inflammatory, M1-phenotype, and increased infiltration and activation of B cells, contributing to a more robust immune response. In vitro, semaglutide stimulated the production of pro-inflammatory cytokines TNF-α, IFN-γ, IL-10, and IL-1β after 24 h of incubation compared to untreated cells. This study for the first time demonstrated the effects of semaglutide on antitumor immunity in a murine model of breast carcinoma.

### 6.12. GLP-1RAs Clinical Studies

While preclinical studies provide evidence for the anticancer effects of GLP-1RAs in breast cancer, clinical data remain limited. The ongoing Phase II clinical trial NCT06518837 aims to evaluate the role of tirzepatide, a dual GLP-1 and Gastric Inhibitory Polypeptide (GIP) receptor agonist, in patients with HR+HER2- breast cancer receiving adjuvant treatment. The primary objective is to determine the proportion of patients achieving a clinically meaningful weight reduction of ≥5% from baseline, a benchmark associated with improved metabolic health and potentially enhanced cancer outcomes. Secondary endpoints include 3-year IDFS and distant relapse-free survival, as well as changes in BMI, fat distribution, metabolic biomarkers, and circulating tumor DNA (ctDNA). Safety, tolerability, and feasibility, measured by treatment discontinuation and completion rates, will also be assessed. The incorporation of exploratory biomarkers, such as ctDNA and adiposity measures, allows the trial to provide mechanistic insights into how metabolic interventions may interact with tumor biology.

The TRIM-EBC trial (NCT06517212) is an ongoing study evaluating tirzepatide-induced weight loss as a strategy to improve outcomes in patients with early-stage, high-risk breast cancer. Specifically, the trial is enrolling individuals with HR+HER2- and node-positive (N+) early breast cancer who are overweight or obese and who have evidence of minimal residual disease (MRD), as detected by ctDNA. The central hypothesis is that pharmacologically induced weight loss with tirzepatide will trigger favorable metabolic and hormonal changes that impair the growth and survival of micrometastatic disease, thereby promoting ctDNA clearance and reducing progression to overt metastasis. The primary endpoints are (1) clearance of plasma ctDNA within two years of documented positivity at study entry and (2) the proportion of patients who remain alive and free of distant metastatic disease two years after ctDNA detection. By targeting obesity-related metabolic drivers of breast cancer recurrence, this trial has the potential to establish a new paradigm in which metabolic modulation complements standard oncologic therapies to improve long-term disease control in a high-risk patient population.

**Figure 1:**
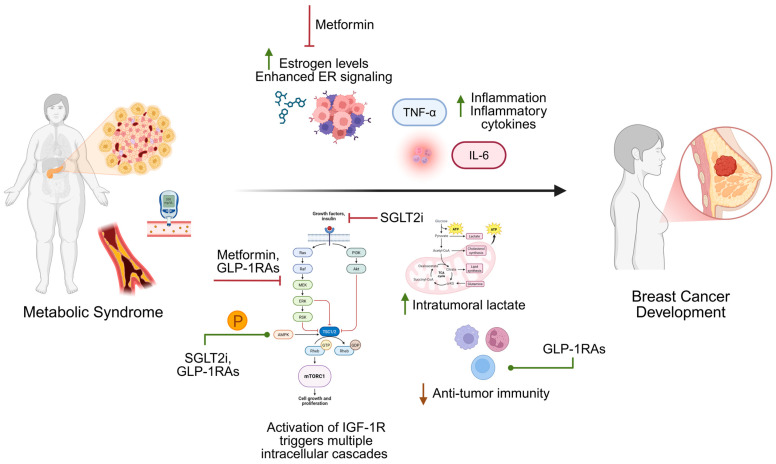
Schematic Illustrating the Intersection of Metabolic Syndrome and Breast Cancer Development.

A summary of completed and ongoing clinical trials evaluating the effect of anti-diabetic therapies in breast cancer is summarized in Table 1 [78,79,81,82,103,104,105,106]. A schematic depicting the intersection of metabolic syndrome and breast cancer development, highlighting key signaling pathways and mechanisms of action of anti-diabetic medications, is shown in Figure 1.

Key components of metabolic syndrome, including obesity, insulin resistance, dyslipidemia, and chronic inflammation, promote tumor initiation and progression through alterations in insulin/IGF-1 signaling and major oncogenic pathways PI3K/AKT/mTOR and Ras/Raf/MEK/ERK, estrogen production, glucose metabolism reprogramming, and immune modulation. The diagram also highlights proposed mechanisms by which anti-diabetic agents, such as GLP-1RAs, SGLT2i, and metformin, may counteract these processes to inhibit tumor growth and improve therapeutic response. Abbreviations: ER = estrogen receptor; SGLT2i = sodium–glucose co-transporter 2 inhibitors; GLP-1RAs = glucagon-like peptide-1 receptor agonists; *p* = phosphorylation; IGF-1R = insulin-like growth factor 1 receptor; TNF-α = tumor necrosis factor-α; IL-6 = interleukin-6. Created in BioRender.com.

## 7. Limitations of Metabolic Therapy in Breast Cancer

Despite promising preclinical data, the therapeutic application of metabolic therapies such as GLP-1RAs and SGLT2 inhibitors in breast cancer remains preliminary. Few oncology-specific clinical trials have been conducted, and existing safety and efficacy data do not yet justify broad clinical application. One potential limitation to consider is their side effect profile. GLP-1RAs frequently cause gastrointestinal adverse effects and warrant careful monitoring, particularly when combined with chemotherapy, which can further worsen dehydration and impair oral intake [107]. Moreover, GLP-1RAs may have off-target effects, variable bioavailability in tumor tissues, and potential interactions with other therapies that require careful evaluation [108]. SGLT2 inhibitors can increase the risk of genitourinary infections, which complicates peri-treatment management and requires careful patient selection and monitoring [109]. The heterogeneity of patient metabolic phenotypes and breast cancer subtypes also introduces substantial complexity. Future studies are needed to evaluate metabolic therapies in well-designed clinical trials before definitive conclusions can be established.

## 8. Conclusions

Metabolic dysregulation is increasingly recognized as a driver of breast cancer pathogenesis, influencing not only tumor-intrinsic signaling but also shaping the immune microenvironment through metabolism-immune interactions. Aberrant glucose and lipid metabolism can alter immune cell composition and function, such as T cell exhaustion, macrophage polarization, and fibroblast activation, to support tumor progression and immune evasion. Recognizing this metabolic-immune axis is critical for understanding disease biology and identifying new therapeutic targets.

Although preclinical studies provide compelling evidence that anti-diabetic and anti-obesity agents can modulate these pathways and potentially enhance antitumor immunity and therapeutic response, clinical validation remains limited and mixed. Large, randomized clinical trials of agents such as metformin have yielded inconsistent results, highlighting the challenges in translating metabolic interventions from the bench to the bedside. Moreover, the well-recognized gap between preclinical efficacy and successful oncologic drug development warrants cautious interpretation of these findings. Future research should prioritize identifying patient subgroups most likely to benefit, integrating metabolic strategies with standard and immunotherapeutic approaches and leveraging biomarkers such as circulating metabolites and immune signatures to optimize clinical trial design. Rigorous translational and clinical studies will be essential to determine whether targeting the metabolic-immune axis can meaningfully improve outcomes for patients with breast cancer.

## Figures and Tables

**Table 1:** List of Clinical Trials Evaluating the Effect of Anti-diabetic Therapies in Breast Cancer.

NCT ID	Reference	Patient Population	Therapy	Phase	Number of Patients Enrolled	Major Clinical Findings	Metabolic Benefit	Status
NCT01589367	Kim et al. (2014) [81]	Postmenopausal ER+ early-stage BC, neoadjuvant setting	Letrozole ± metformin	Phase II	208	Overall clinical response rate of 61.4%, no statistical significance between the metformin + letrozole vs. placebo + letrozole cohort (66.7% versus 56.4%, *p* = 0.193).	N/A	Completed
NCT01980823	—	Newly diagnosed operable breast cancer (“window of opportunity”)	Metformin + atorvastatin	Early Phase I	23	Determine if dual combination treatment significantly impacts Ki-67 proliferation index pre-surgery, results pending	N/A	Completed
NCT00930579	Kalinsky et al. (2015) [103]	Newly diagnosed DCIS or stage I-III early invasive BC in non-diabetic women with BMI ≥ 25	Metformin	Phase II (pre-surgical)	35	No change in Ki-67 proliferation index	Significant improvement in BMI and reductions in leptin and cholesterol with metformin	Completed
NCT01793948	—	Overweight/obese women at elevated breast cancer risk	Metformin vs. placebo	Phase 0	24	Biomarker analysis in prevention setting, results pending	N/A	Completed
NCT01340300	Meyerhardt et al. (2019) [104]	Stage I-III breast or colorectal cancer survivors post adjuvant treatment	Exercise ± metformin	Phase II	139	N/A (primary endpoint is metabolic profile)	Both exercise and metformin led to significantly reduced levels of fasting insulin and improvements in BMI compared to the control group	Completed
NCT01101438	Goodwin et al. (2022) [79]	Early-stage BC, adjuvant setting	Metformin vs. placebo	Phase III	3649	No improvement in IDFS; exploratory analyses show that metformin use was associated with longer IDFS and OS in HER2+ BC	N/A	Completed
NCT02028221	Tapia et al. (2021) [78]	Premenopausal women with BMI ≥ 25 at risk for BC	Metformin vs. placebo	Phase II	151	Compared to placebo, metformin did not change % breast density and dense breast volume but led to a numerical but not significant decrease in non-dense breast volume	Favorable changes in measures of adiposity and borderline breast density reduction in metformin group	Completed
NCT01310231	Pimentel et al. (2019) [105]	Metastatic breast cancer in non-diabetic women	Standard chemotherapy + metformin	Phase II	40	No significance found on response rate, PFS, or OS	N/A	Completed
NCT06763328	—	ER- stage I–III invasive BC	Metformin	Phase III	200	Determine if metformin can prevent or reverse insulin resistance in BC patients after chemotherapy, results pending	Results pending	Recruiting
NCT04248998	Ligorio et al. (2025) [82]	Localized TNBC	Cyclic fasting-mimicking diets ± metformin	Phase II	30	Overall pCR rate of 56.6% was markedly higher than the historical range of 26–39% reported in prior Phase II/III neoadjuvant chemotherapy trials	N/A	Active, not recruiting
NCT05023967 (TEAM study)	—	Non-diabetic patients with early-stage breast cancer (prevention/biomarker setting)	Time-restricted eating (nightly fasting) + extended-release metformin vs. control	Phase IIb	120	Determine whether nightly fasting + metformin reduces tumor cell proliferation and related biomarkers (e.g., Ki-67)	Results pending	Recruiting
NCT01042379 (I-SPY Trial)	Yee et al. (2021) [106]	High-risk stage II/III HER2- operable breast cancer (neoadjuvant platform; multiple experimental arms)	One reported arm: Paclitaxel + ganitumab + metformin (PGM) → AC vs. paclitaxel → AC (SOC)	Phase II (adaptive platform)	5000	PGM showed numerically higher pCR in HR-/HER2- patients (32% vs. 21%) but did not meet graduation thresholds; no EFS improvement	N/A	Recruiting; PGM arm completed within platform
NCT05090358	—	HR+HER2-, PIK3CA-mutant metastatic BC on alpelisib + fulvestrant	Canagliflozin vs. diet	Phase II	15	Primary endpoint: proportion of patients who remain free from grade 3–4 hyperglycemia at 12 weeks; secondary endpoints: ORR and PFS at 6 and 12 months. Results pending.	Results pending	Active, not recruiting
NCT05989347	—	Early-stage HER2- BC in hyperinsulinemic women	Dapagliflozin + neoadjuvant therapy	Phase I	20	Assess changes in fasting plasma glucose and insulin levels as surrogate markers for insulin resistance. Results pending.	Results pending	Recruiting
NCT06304857	—	Non-diabetic patients with early-stage invasive BC receiving anthracyclines	Dapagliflozin vs. placebo	Phase III	188	Evaluate whether dapagliflozin can prevent anthracycline-related cardiotoxicity	Results pending	Recruiting
NCT05025735	—	PIK3CA-mutant metastatic HR+HER2- BC	Dapagliflozin + alpelisib + fulvestrant	Phase II	25	Evaluate whether the addition of dapagliflozin to alpelisib and fulvestrant leads to significant reduction in all-grade hyperglycemia	Results pending	Recruiting
NCT06518837	—	HR+HER2- early-stage BC patients receiving adjuvant treatment	Tirzepatide, a dual GLP-1/GIP receptor agonist	Phase II	40	Primary objective is to determine the proportion of patients achieving a clinically meaningful weight reduction of ≥5% from baseline; secondary endpoints include 3-year IDFS and changes in BMI, fat distribution, metabolic biomarkers, and ctDNA	Results pending	Recruiting
NCT06517212	—	Overweight or obese patients with early-stage, HR+HER2-, node-positive breast cancer and detectable minimal residual disease via ctDNA	Tirzepatide	Phase II	48	Aims to assess whether tirzepatide-induced weight loss leads to metabolic, hormonal, and immunologic changes that result in clearance of ctDNA and reduce progression to overt metastasis	Results pending	Recruiting

Abbreviations: ER = estrogen receptor; BC = breast cancer; DCIS: ductal carcinoma in situ; BMI = body mass index; IDFS = invasive disease-free survival; OS = overall survival; PFS = progression-free survival; pCR = pathological complete response; HR = hormone positive; HER2 = human epidermal growth factor receptor 2; PIK3CA = phosphatidylinositol-4,5-bisphosphate 3-kinase catalytic subunit alpha; ctDNA = circulating tumor DNA; AC = Adriamycin/Cytoxan; SOC = standard of care; EFS = event-free survival.

## Data Availability

Not applicable.
